# Esophageal Rupture and Mediastinitis Following Blakemore Tube Tamponade: A Cardiothoracic Emergency and Protocol-Based Prevention

**DOI:** 10.7759/cureus.83097

**Published:** 2025-04-27

**Authors:** Rola A Ali, Jordan A Lebron, Peter F Houmann, Dwayne Gordon

**Affiliations:** 1 Internal Medicine, AdventHealth Orlando, Orlando, USA

**Keywords:** balloon tamponade, esophageal rupture, esophago gastroduodenoscopy, sengstaken-blakemore tube, severe hematemesis

## Abstract

The Sengstaken-Blakemore tube (SBT) is a balloon tamponade system employed as a temporary measure to achieve short-term hemostasis in patients experiencing uncontrolled bleeding from gastroesophageal varices. While these tubes are generally well-tolerated, they carry a risk of significant complications, including mucosal necrosis, aspiration pneumonia, esophageal perforation, and rebleeding after deflation of the balloon. Among these, esophageal perforation is a particularly rare but life-threatening complication that may result from improper placement or over-inflation of the balloon. This report presents a rare case of Blakemore tube-induced esophageal perforation, resulting from balloon misplacement, in a patient receiving treatment for variceal hemorrhage.

The objectives of this case report are to highlight the potential for serious complications arising from improper tube placement and to provide guidance on preventing such incidents. This report emphasizes the need for monitoring of balloon tamponade devices and verification of proper tube positioning before and after the inflation of the gastric balloon, especially in emergency settings where rapid intervention is essential. By outlining the clinical presentation, diagnostic challenges, and treatment strategies in this case, we aim to enhance clinician awareness of the rare but serious risks associated with SBT use and provide practical guidance for minimizing complications, improving patient outcomes, and ensuring the safety and effectiveness of this life-saving procedure.

## Introduction

Esophageal perforation in adults is a rare but serious condition, often resulting in high morbidity and mortality. Iatrogenic injury is the most common cause, accounting for approximately 70% of esophageal perforations [1]. Balloon tamponade is a widely used endoscopic technique for achieving short-term hemostasis in patients with uncontrolled esophageal variceal bleeding, serving as a bridge to definitive treatments such as transjugular intrahepatic portosystemic shunt (TIPS). One such device, the Sengstaken-Blakemore tube (SBT), is inflated within the gastric lumen to tamponade the gastroesophageal junction. While effective in controlling bleeds, it carries significant risks, including mucosal injury, necrosis, tube migration, bronchopulmonary aspiration, rebleeding, and, most critically, esophageal perforation. Balloon misplacement, often due to improper positioning or overinflation, can exert excessive pressure on the esophageal wall, leading to localized ischemia and mechanical injury, which may progress to perforation [2]. We herein report a case of esophageal perforation induced by Blakemore tube misplacement.

## Case presentation

A 69-year-old male patient with a past medical history of nonalcoholic steatohepatitis (NASH) cirrhosis, complicated by portal hypertension with associated ascites and gastro-esophageal varices requiring banding four months ago, presented as an outside hospital transfer with a one-day history of massive hematemesis. Vital signs at the prior institution included a blood pressure of 93/75 mm Hg, heart rate of 136 bpm, temperature of 96.9°F, respiratory rate of 18, and oxygen saturation of 100%. The patient was alert but physically exhibited pallor, restlessness, and jaundice. Lab tests revealed a hemoglobin of 5 g/dL, white blood cell count of 16,030 cells/mL, and albumin of 2.2 g/dL. Other lab values included bilirubin 6 mg/dl, INR 1.69, prothrombin time 20 sec, ammonia 42 umol/l, and glucose 261 mg/dL. The patient was placed on a massive transfusion protocol and initiated on antibiotics and continuous infusion of octreotide and protonix. Due to ongoing hematemesis, endotracheal intubation was performed for airway protection, and the patient was transferred to the intensive care unit for further management.

Emergent endoscopy (EGD) revealed large (>5 mm) varices in the middle third of the esophagus. Six bands were successfully placed, though the varices were not completely eradicated. Bleeding continued, with red blood throughout the examined stomach, and hematin was found in the ampulla, duodenal bulb, and the first and second portions of the duodenum. Hemospray was attempted at the gastroesophageal junction, but due to the copious blood, it malfunctioned after a few seconds.

The patient became hemodynamically unstable, with a stat hemoglobin of 4.6 g/dl drawn during the procedure. A Blakemore tube was placed for hemostasis and balloon tamponade. At 45 cm from the incisors, the gastric balloon was filled with 300 cc of air, clamped, and pulled tight to the gastroesophageal junction. Suctioning of the gastric lumen showed minimal blood return, which then ceased. The gastric balloon and Blakemore tube were tightly secured to the endotracheal tube at the 24 cm mark from the incisors.

**Figure 1:**
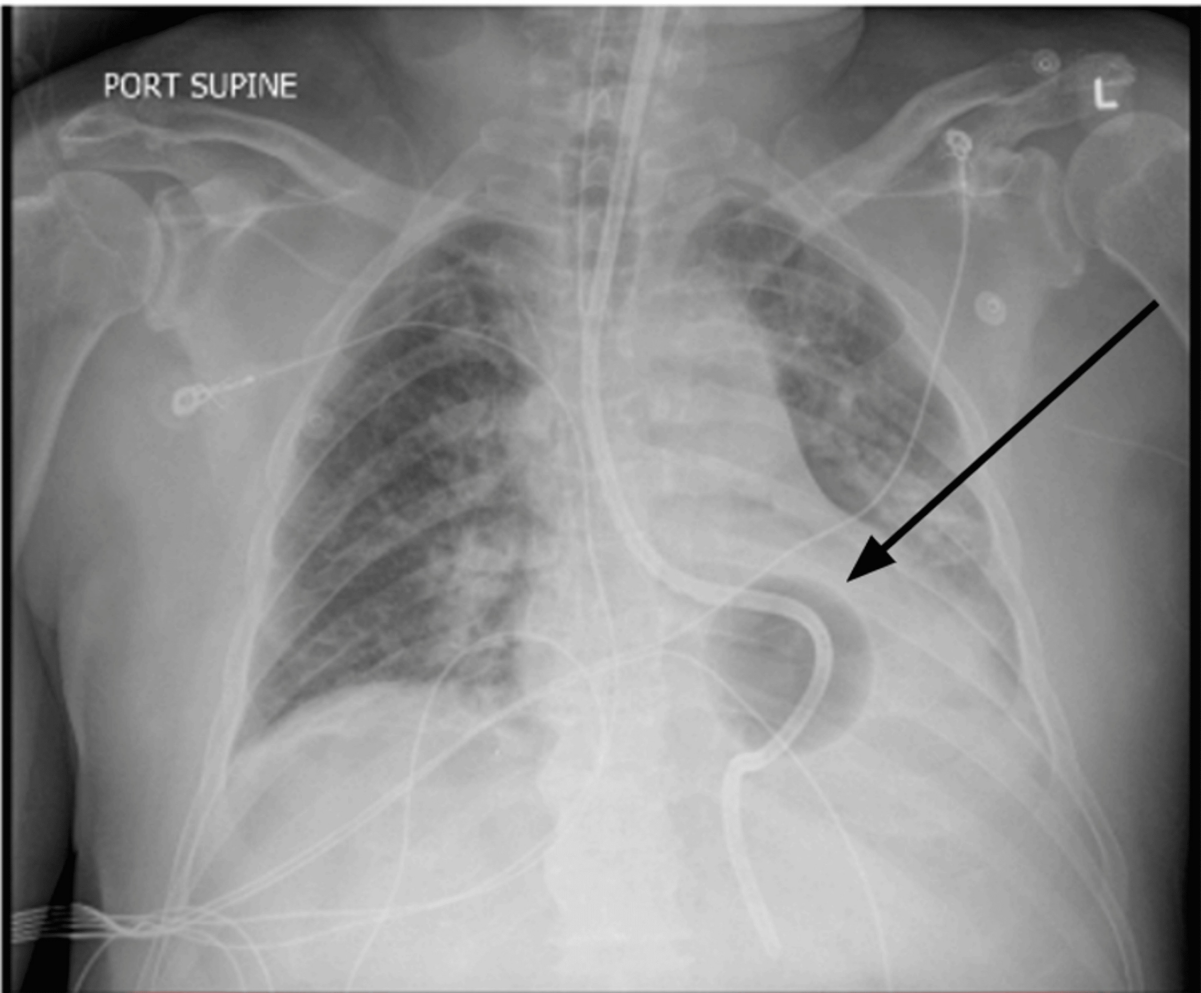
Chest x-ray showing malpositioned Blakemore catheter

**Figure 2:**
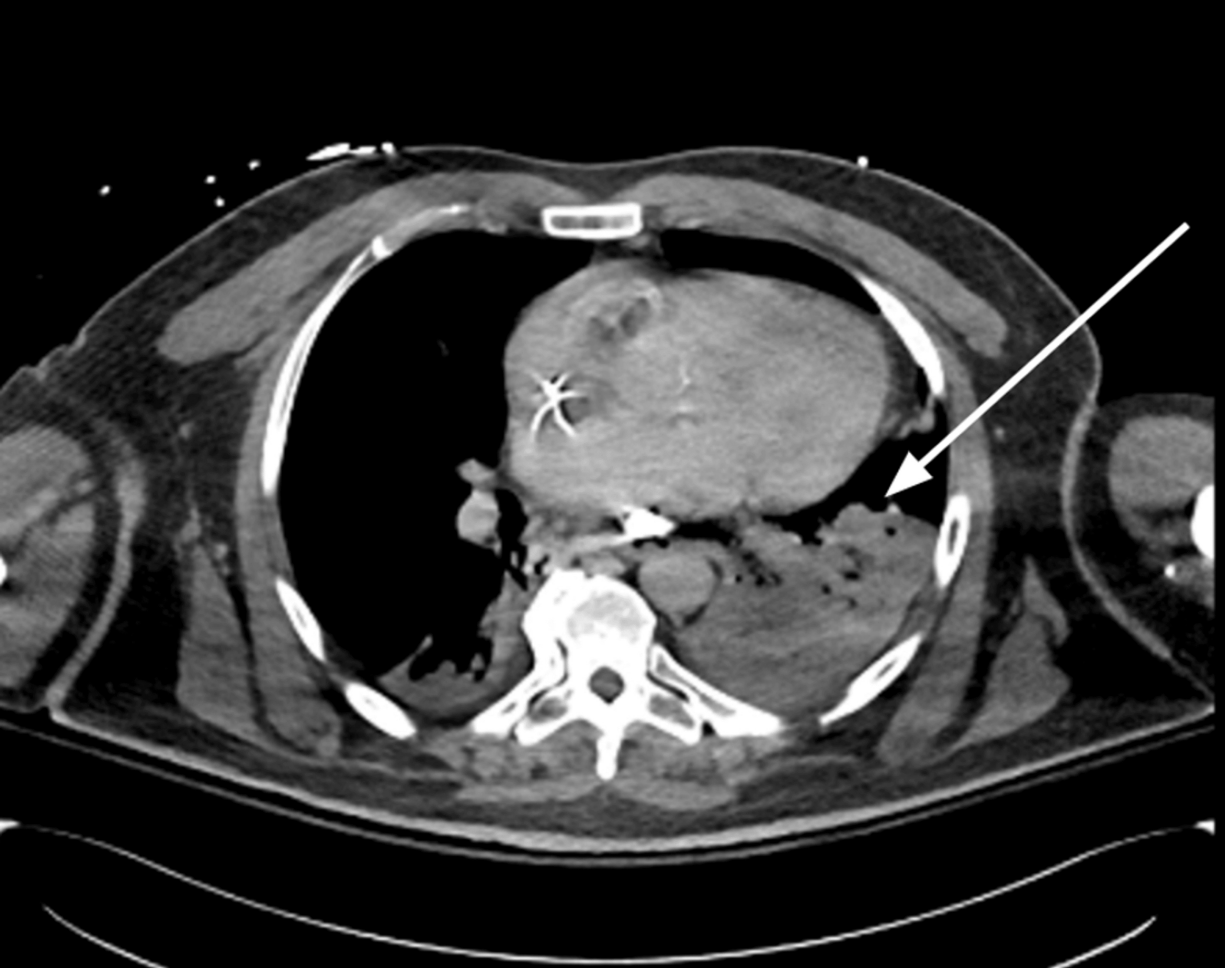
CT chest with IV contrast showing the Blakemore catheter has perforated the distal esophagus and is malpositioned and insufflated in the left lower lobe (LLL) with associated small left pneumothorax and complete LLL consolidation

**Figure 3:**
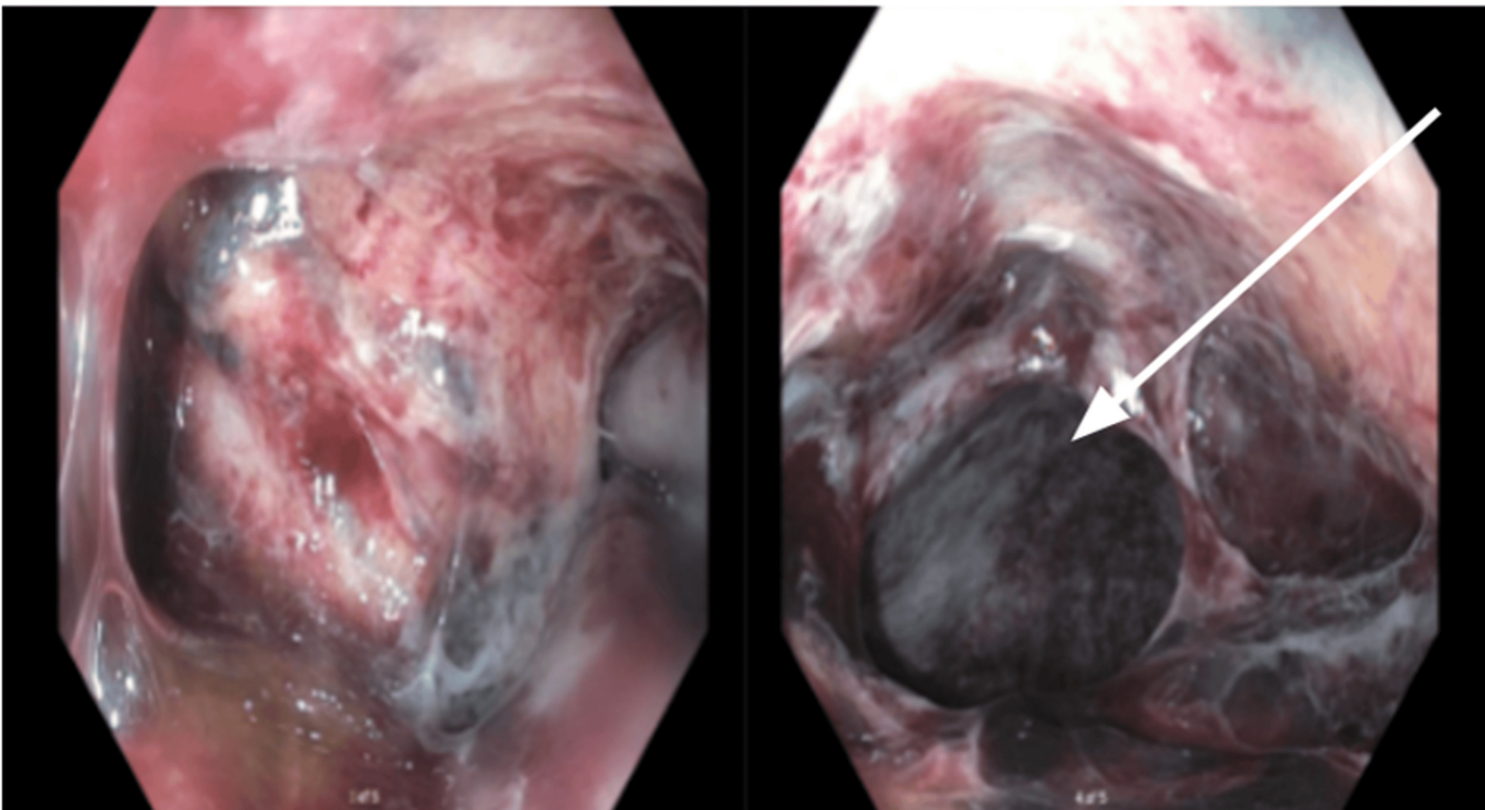
Endoscopic views revealing a large esophageal defect with the lung visible through the perforation area.

The patient received multiple red blood cell and fresh frozen plasma transfusions for hemodynamic stability and was transferred emergently to our facility for an emergent TIPS procedure. Chest X-ray (Figure 1) showed a malpositioned Blakemore catheter with bilateral basilar infiltrates. Computed tomography (CT) chest with contrast (Figure 2) revealed distal esophageal perforation caused by the malpositioned Blakemore tube, which was insufflated in the left chest cavity, resulting in a left pneumothorax and complete left lower lobe consolidation. The patient underwent a successful emergent TIPS procedure, with a reduction in portal pressure gradient from 30 mmHg to 13 mmHg. Emergent endoscopy (Figure 3) was performed, revealing a large perforation located 36 to 40 cm from the incisors. The perforation was significant, with the lung visible through the defect. The Blakemore tube's gastric balloon was deflated and withdrawn without complications. 

The Center for Interventional Endoscopy (CIE) team was consulted for endoscopic stenting to seal the mucosal defect and restore esophageal continuity. However, due to the large size of the perforation, the patient was deemed ineligible for this approach, and cardiothoracic surgery was consulted for surgical management. He was taken to the operating room and underwent an exploratory laparotomy, modified Ivor Lewis esophagectomy, and left posterolateral thoracotomy. The procedure included complete decortication and drainage with debridement of the left pleural space for empyema, ligation of multiple extensive perihilar and paraesophageal varices throughout the chest, and placement of a feeding jejunostomy tube. Postoperatively, he experienced ventilator-dependent respiratory failure but was successfully extubated following aggressive pulmonary hygiene. However, he developed sepsis that progressed to septic shock despite treatment with vancomycin, meropenem, and micafungin. Repeat EGD one month later showed the esophago-gastric anastomosis had an intact staple line with no evidence of bleeding, dehiscence, or abnormalities at the surgical anastomosis. The patient's condition continued to deteriorate. He developed hepatic encephalopathy and worsening volume overload due to hepatorenal syndrome despite receiving albumin, octreotide, and continuous renal replacement therapy (CRRT). Given his multiorgan failure, the patient was deemed not to be a suitable candidate for liver transplantation. Given the grave prognosis, a multidisciplinary discussion took place with the family, who opted to pursue comfort care. The patient passed away a week later.

## Discussion

Acute variceal bleeding is a feared sequelae of liver cirrhosis and has a high mortality rate, reported as high as 20% in some studies [3]. The acute management of upper gastrointestinal bleeding from a suspected variceal source consists of initial, temporizing measures and definitive measures. Temporizing measures include fluid resuscitation to maintain hemodynamic stability, red blood cell transfusion, correction of coagulopathy with other blood products, and administration of antibiotics, which have been shown to reduce the risk of rebleeding, mortality, and superimposed infections like spontaneous bacterial peritonitis [4]. Additionally, vasoactive medications as terlipressin or octreotide are given to reduce portal pressures. Definitive management of variceal bleeding is achieved through a variety of endoscopic methods, including sclerotherapy, banding, cyanoacrylate glue, and coil embolization, as well as the TIPS procedure [5].

Balloon tamponade using a Sengstaken-Blakemore tube is another temporizing measure that is less frequently used due to its complication rates and severity of its potential complications. This procedure is generally reserved for patients with severe hemodynamic instability that is unresponsive to initial resuscitative measures until definitive management like TIPS can be instituted. Complications of Blakemore tube placement arise in around 14% of patients and can include rebleeding after balloon deflation, esophageal perforation, mucosal pressure necrosis, and aspiration pneumonia [2]. We described a case of esophageal rupture with subsequent massive pneumothorax due to balloon misplacement and gastric balloon inflation in the distal esophagus.

Given the emergent situation that may evolve with uncontrolled variceal hemorrhage, proper protocols of use, including the use of radiographic imaging to ensure proper balloon positioning below the level of the diaphragm, may be overlooked. To reduce the risk of esophageal perforation when using a Blakemore tube, several precautions should be taken. After inserting the tube to 50 cm at the incisors, the stomach should be auscultated while injecting air through the tube. This can provide an initial indication that the tube is in the stomach, allowing one to inflate the balloon with 40-50 cc of air. However, it is crucial to confirm the positioning of the gastric balloon with radiography before fully inflating it. Once verified, the gastric balloon can then be inflated with 300-400 cc of air. Maintaining adequate tension on the tube and securing its position is essential for effective hemostasis when the gastric balloon is fully inflated [6].

The esophageal balloon should only be inflated if the properly positioned gastric balloon does not achieve adequate hemostasis. In such cases, the esophageal balloon should be inflated to a maximum of 30-45 mmHg, and the pressure should be verified at least every hour. Proper training of clinical staff in the use of these devices is critical, as failure to frequently and accurately monitor the pressures can significantly increase the risk of esophageal necrosis [7].

Esophageal rupture following Blakemore tube misplacement carries an extremely high risk of morbidity and mortality. To date, there remain no specific guidelines on definitive management of this complication, and ideal treatment remains controversial [1]. Generally, patients are kept strictly NPO (nothing by mouth) and provided parenteral nutritional support. Intravenous fluid resuscitation is administered, along with close monitoring and correction of electrolyte imbalances. Given the high risk of bacterial translocation to the mediastinal cavity, which may result in mediastinitis and sepsis, broad-spectrum antibiotics are initiated, often alongside empiric antifungal therapy, usually targeting *Candida* species. Abscesses or fluid collections may need to be drained. If the perforation has a contained leak, medical management may be sufficient, provided that experienced medical staff is available for invasive intervention in cases of clinical deterioration or hemodynamic compromise. Both endoscopic and open surgical management have been performed in these cases, and the choice of method will depend on the physician's judgment [8]. Surgical options may include debridement with primary repair, temporary diversion with delayed reconstruction, or esophagectomy. The choice of repair depends on several factors, including patient stability, etiology of the rupture, failed prior closures with ongoing sepsis, underlying malignancy, presence of necrosis, or esophageal motility disorders. Esophageal stenting is a very promising intervention, often helping to provide leak occlusion at a high rate and allowing for the maintenance of oral nutrition [9]. In our case, the patient was treated with open surgery due to the large size of the esophageal defect; he survived the esophagectomy and thoracotomy, and follow-up endoscopy demonstrated an intact esophagogastric anastomosis. 

## Conclusions

This case study highlights the critical need for adhering to proper protocols, including X-ray imaging, when employing temporizing balloon tamponade for acute esophageal variceal bleeding. Improper use of a Blakemore tube can result in catastrophic complications, including esophageal rupture, particularly in patients already compromised by significant variceal hemorrhage. Preventive strategies, such as annual training and review of proper Blakemore tube technique, can help mitigate these risks. Strict adherence to procedural guidelines is essential to minimize patient harm and avoid life-threatening outcomes, as this case illustrates.
